# Integrin αvβ3 Engagement Regulates Glucose Metabolism and Migration through Focal Adhesion Kinase (FAK) and Protein Arginine Methyltransferase 5 (PRMT5) in Glioblastoma Cells

**DOI:** 10.3390/cancers13051111

**Published:** 2021-03-05

**Authors:** Pulin Che, Lei Yu, Gregory K. Friedman, Meimei Wang, Xiaoxue Ke, Huafeng Wang, Wenbin Zhang, Burt Nabors, Qiang Ding, Xiaosi Han

**Affiliations:** 1 Department of Anesthesiology & Perioperative Medicine, Division of Molecular and Translational Biomedicine, University of Alabama at Birmingham, Birmingham, AL 35294, USA; pche@uab.edu (P.C.); meimei1@uab.edu (M.W.); 2 Guiyang Maternal and Child Health Hospital, Guiyang 550001, China; 775438959@qq.com; 3 Department of Pediatrics, University of Alabama at Birmingham, Birmingham, AL 35294, USA; GFriedman@peds.uab.edu; 4 State Key Laboratory of Silkworm Genome Biology, Southwest University, Chongqing 400716, China; kexiaoxue@swu.edu.cn; 5 Department of Neurology, University of Alabama at Birmingham, Birmingham, AL 35294, USA; huafengwang@uabmc.edu (H.W.); zhangwenbin1978@126.com (W.Z.); bnabors@uabmc.edu (B.N.); 6 School of Life Science, Shanxi Normal University, Linfen City 041004, China

**Keywords:** integrin, metabolism, focal adhesion kinase, protein arginine methyltransferase 5, glioblastoma, migration, invasion, proliferation, cancer

## Abstract

**Simple Summary:**

Interactions of integrins with the extracellular matrix play a key role in cancer cell migration, invasion, and growth. However, whether integrin engagement promotes cancer progression through metabolic reprogramming has not been completely understood. This study investigates the role and mechanism of integrin αvβ3 engagement with its ligand in metabolic reprogramming. The data support that integrin αvβ3 plays an important role in increased glucose uptake and aerobic glycolysis, as well as in decreased mitochondrial oxidative phosphorylation, in glioblastoma cells. In addition, the data imply that focal adhesion kinase (FAK) and protein arginine methyltransferase 5 (PRMT5) are likely downstream effectors of integrin αvβ3, and regulate metabolic shift toward glycolysis. These findings provide new insight into how cancer cells regulate their metabolism based on microenvironmental cues transmitted by integrin and extracellular matrix proteins, and how the signals eventually translate to metabolic modifications coupled with changes in cell behavior, including migration, invasion, and growth.

**Abstract:**

Metabolic reprogramming promotes glioblastoma cell migration and invasion. Integrin αvβ3 is one of the major integrin family members in glioblastoma multiforme cell surface mediating interactions with extracellular matrix proteins that are important for glioblastoma progression. The role of αvβ3 integrin in regulating metabolic reprogramming and its mechanism of action have not been determined in glioblastoma cells. Integrin αvβ3 engagement with osteopontin promotes glucose uptake and aerobic glycolysis, while inhibiting mitochondrial oxidative phosphorylation. Blocking or downregulation of integrin αvβ3 inhibits glucose uptake and aerobic glycolysis and promotes mitochondrial oxidative phosphorylation, resulting in decreased migration and growth in glioblastoma cells. Pharmacological inhibition of focal adhesion kinase (FAK) or downregulation of protein arginine methyltransferase 5 (PRMT5) blocks metabolic shift toward glycolysis and inhibits glioblastoma cell migration and invasion. These results support that integrin αvβ3 and osteopontin engagement plays an important role in promoting the metabolic shift toward glycolysis and inhibiting mitochondria oxidative phosphorylation in glioblastoma cells. The metabolic shift in cell energy metabolism is coupled to changes in migration, invasion, and growth, which are mediated by downstream FAK and PRMT5 in glioblastoma cells.

## 1. Introduction

Integrins are a large family of heterodimeric transmembrane glycoprotein receptors, which mediate cell adhesion to a wide variety of extracellular matrix (ECM) proteins. In glioblastomas, integrin αvβ3 is consistently overexpressed [1,2]. Detailed analysis by immunohistochemistry and in situ hybridization revealed that αvβ3 is associated with high-grade gliomas, including anaplastic astrocytomas and glioblastoma multiforme (GBM), but is not detected in the vasculature of low-grade gliomas or in normal tissue [3], suggesting that this integrin might be associated with tumor progression [2]. Interestingly, expression of αvβ3 is also observed at the periphery of glioblastomas and is frequently expressed with metalloprotease-2 (MMP-2) in tumor cells at the invading front [4,5,6]. These data suggest that αvβ3 may play an important role in promoting GBM cell invasion, growth, and tumor formation.

The ECM ligands that are frequently recognized by αvβ3 integrin include osteopontin, vitronectin and others [1]. Osteopontin, a member of the small integrin-binding ligand N-linked Glycoprotein family [7], is expressed in normal mineralized tissues, epithelial cells of some metabolically active ducts and many neoplastic tissues [8]. Several studies have reported that osteopontin is overexpressed in glioblastoma with higher expression in GBM compared to low-grade brain tumors [9]. Furthermore, osteopontin is highly expressed in the microvasculature of GBMs and has been implicated in malignant glioma invasion and angiogenesis [10,11]. High serum osteopontin levels were also demonstrated to be a poor prognosis marker in GBM patients [12]. Inhibition or knockdown of osteopontin significantly inhibited GBM formation in vivo [13,14].

Most cancers, including GBM, predominantly rely on aerobic glycolysis instead of oxidative phosphorylation (OXPHOS) to generate ATP [15,16], resulting in the partial oxidation of glucose to pyruvate and its conversion to lactate, even in the presence of physiological oxygen levels. This phenomenon is known as the Warburg effect [17]. Increased glycolytic metabolism is shown to be important for cellular functions by enhancing the supply of NAD+, NADPH, and other essential molecules for biomass synthesis [18,19,20]. Blocking glycolysis inhibits glioblastoma cell growth and tumor formation in animal models [21,22].

The role of αvβ3 integrin in the metabolic shift toward glycolysis in glioblastoma has not been well investigated. Because integrin binding to extracellular matrix and activating focal adhesion kinase (FAK) following integrin engagement are important for cell growth and tumor formation [11,23], we hypothesized that integrin αvβ3 engagement may initiate signaling through FAK that results in a metabolic shift towards glycolysis and promotes glioblastoma cell invasion and growth. We investigated the effect of αvβ3 integrin and ECM osteopontin engagement on glucose metabolism in GBM. We found that the binding of αvβ3 integrin and osteopontin plays a critical role in increasing glucose uptake and glycolysis, while concomitantly reducing mitochondria OXPHOS in glioblastoma cells. Knockdown of αv or β3 integrin, inhibition of αvβ3 binding, or inhibition of FAK signaling, inhibited the metabolic shift. We have previously shown that protein arginine methyltransferase 5 (PRMT5) expression correlates with grade in malignant glioma [24], and PRMT5 knockdown decreased glycolysis and increased OXPHOS in glioblastoma cells. These results support that integrin αvβ3 engagement with osteopontin initiates an important signaling cue driving metabolic reprogramming, which promotes glioblastoma cell migration, invasion, and growth.

## 2. Results

### 2.1. Integrin αvβ3 Plays an Important Role in Metabolic Reprogramming toward Glycolysis in Glioblastoma (GBM) Cells

**Figure 1:**
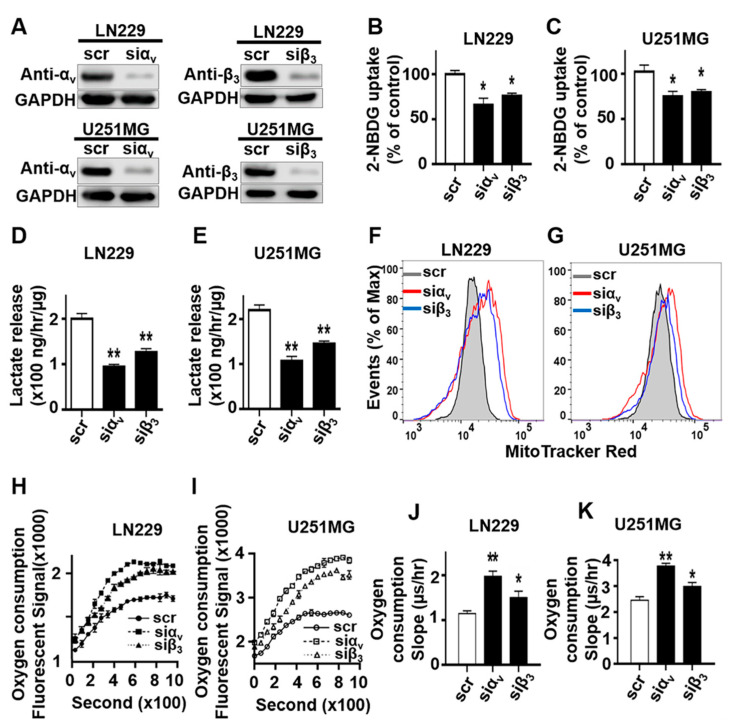
Integrin αvβ3 knockdown inhibits glucose uptake and lactate production but promotes mitochondria function and O_2_ consumption in LN229 and U251MG cells. (**A**) Western blot result of siRNA-mediated knockdown of αv or β3. A scrambled siRNA (scr) was used as the control for the effects of αv or β3 knockdown. (**B**,**C**) Uptake of glucose analog 2-NBDG was reduced in cells with αv or β3 knockdown. (**D**,**E**) Lactate levels in culture medium decreased in cells with αv or β3 knockdown. (**F**,**G**) Flow cytometry analysis of MitoTracker stained cells indicate increased mitochondria function in cells with αv or β3 knockdown. (**H**,**I**) Oxygen consumption curve shifted higher in cells with αv or β3 knockdown, suggesting increased oxygen consumption. Oxygen consumption is represented by the life time signal increased in the presence or absence of antibodies. (**J**,**K**) Cellular oxygen consumption rate (slop) calculated based on the oxygen consumption curve. The oxygen consumption rate is increased in cells with αv or β3 knockdown when compared to controls. Both 2-NBDG uptake and lactate production were normalized against cellular protein. All experiments were repeated at least three times. Data are represented as mean ± standard error (SE), * *p* < 0.05, ** *p* < 0.01.

Cancer cells preferentially use glycolysis for energy production, which generates only 2 ATPs per glucose molecule. Consequently, cancer cells requires significantly more glucose to compensate for energy production. The incomplete metabolism of glucose produces large amount of lactate, the final product in the glycolysis pathway. To elucidate the role of integrin αvβ3 in glioblastoma glucose metabolism, LN229 and U251MG glioblastoma cells were transfected with small interfering RNA (siRNA) directed against integrin αv or β3 subunit respectively. LN229 and U251MG are two commonly used GBM cell lines. Knockdown (over 90%) of αv or β3 subunit was confirmed by Western blot analysis in LN229 and U251MG cells treated with siRNA toward αv (siαv) or β3 (si β3) when compared to control siRNA, respectively (Figure 1A and Figure S1). We utilized 2-(N-(7-Nitrobenz-2-oxa-1,3-diazol-4-yl)Amino)-2-Deoxyglucose (2-NBDG), a florescent glucose analog that is taken up by cells but is not metabolized, to assess glucose uptake. Knockdown of either αv or β3 significantly decreased 2-NBDG uptake and the levels of lactate in the culture medium in both LN229 and U251MG cells grown on osteopontin coated plate (Figure 1B–E). These results demonstrate that αvβ3 knockdown decrease glucose uptake and lactate production.

Because of the Warburg effect, cancer cells rely less on mitochondria oxidative phosphorylation to generate ATPs compares to normal cells [15]. In order to understand the role of integrin αvβ3 in mitochondrial function in GBM cells, we determined whether integrin αvβ3 knockdown affects mitochondrial membrane potential, a parameter reflecting the oxidative phosphorylation status of mitochondria. MitoTracker probe was used to monitor mitochondrial activity as it binds irreversibly to the polarized mitochondrial membrane. The probe possesses a reactive chloromethyl group that forms a covalent bond with thiols on proteins, which traps MitoTracker Red CMXRos probes. The MitoTracker Red CMXRos probes accumulate electrophoretically into mitochondria in response to the highly negative mitochondrial membrane potential [25,26]. The MitoTracker labeled cells were analyzed by flow cytometry. Knockdown of either αv or β3 led to increased fluorescent intensity of MitoTracker labeling in both LN229 and U251MG cells, indicating increased mitochondria function (Figure 1F,G). Next we measured the cellular oxygen consumption, another indicator of mitochondrial oxidative phosphorylation function. Knockdown of either αv or β3 led to significant increases in the rate of oxygen consumption in LN229 and U251MG cells (Figure 1H–K), indicating increased mitochondria function following integrin αvβ3 knockdown. These results strongly support an important role of integrin αvβ3 in metabolic reprogramming by promoting glucose uptake and decreasing mitochondrial function in GBM cells.

### 2.2. Engagement of Integrin αvβ3 with Osteopontin Is Associated with a Metabolic Shift toward Glycolysis in GBM Cells

**Figure 2:**
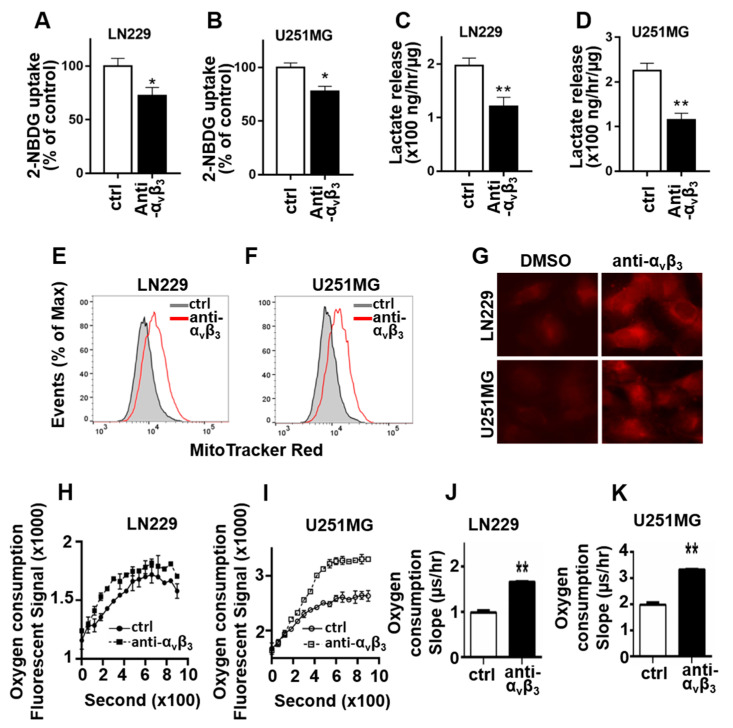
Engagement of integrin αvβ3 with osteopontin promotes glucose uptake and lactate production but inhibits mitochondria function and O_2_ consumption in LN229 and U251MG. (**A**,**B**) Uptake of glucose analog 2-NBDG was reduced in cells treated αvβ3 blocking antibody when compared to cells treated with control isotype antibody (ctrl). (**C**,**D**) Lactate in culture medium was decreased in cells treated with αvβ3 blocking antibody. (**E**,**F**) Flow cytometry analysis of MitoTracker stained cells showed an increase in mitochondrial function in cells treated with αvβ3 blocking antibody. (**G**) Representative immunofluorescent images of the MitoTracker labeled cells with αvβ3 or control antibody (400×). Stronger fluorescence appeared in cells treated with αvβ3 blocking antibody. (**H**,**I**) Oxygen consumption curve increased in cells treated with αvβ3 blocking antibody. (**J**,**K**) Cellular oxygen consumption rate (slop) calculated based on the oxygen consumption curves in panels (**H**,**I**). All experiments were repeated at least three times. Data are represented as mean ± SE, * *p* < 0.05, ** *p* < 0.01.

Because integrin αvβ3 knockdown inhibits glycolysis and promotes mitochondria OXPHOS, we next examined whether αvβ3 integrin engagement with osteopontin is required for regulation of glucose metabolism in GBM cells. We chose to examine αvβ3 and osteopontin engagement on glucose metabolism because their interaction is an important signaling events in GBM tumor invasion and growth [1,2,3,6,11]. LN229 and U251MG GBM cells were plated on osteopontin (10 μg/mL) coated plate in the presence or absence of anti-αvβ3 blocking antibody as described previously [1,11], then glucose uptake, glycolysis, and mitochondrial activity were measured (Figure 2). Glucose uptake was significantly decreased in LN229 and U251MG cells treated with αvβ3 blocking antibody when compared to that in cells treated with control antibody (Figure 2A,B). The lactate levels in the culture medium were significantly lower in LN229 and U251MG cells treated with αvβ3 blocking antibody (Figure 2C,D). In addition, blocking of αvβ3 integrin engagement with osteopontin significantly increased MitoTracker labeling in both LN229 and U251GM cells, indicating enhanced mitochondrial membrane potential and activity (Figure 2E,F). Consistent with the flow cytometry results, imaging of MitoTracker labeled cells showed enhanced fluorescence in cells treated with αvβ3 blocking antibody (Figure 2G). Finally, cellular oxygen consumption rate was significantly increased in both LN229 and U251MG cells treated with αvβ3 blocking antibody (Figure 2H–K). Taken together, these results suggest that engagement of integrin αvβ3 with osteopontin results in a metabolic shift towards glycolysis and inhibits mitochondrial oxidative phosphorylation in GBM cells.

### 2.3. Blockade of FAK Activation Inhibits Glucose Uptake and Glycolysis but Promotes Mitochondrial Function in GBM Cells

**Figure 3:**
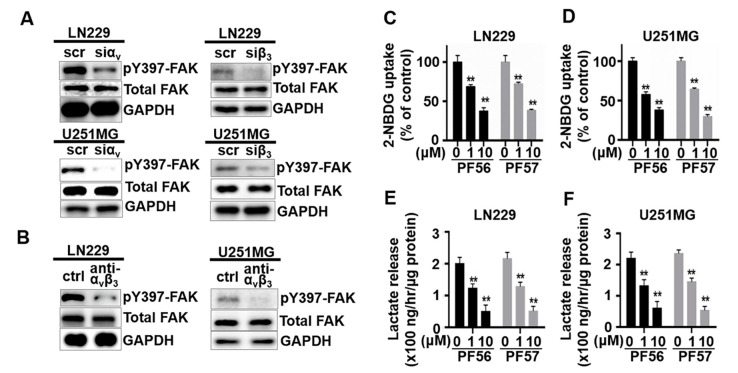
Focal adhesion kinase (FAK) inhibition reduces glucose uptake and lactate acid production. LN229 and U251MG cells were plated on osteopontin and (**A**,**B**) FAK activation is decreased in response to siRNA mediated knockdown of αv or β3 when compared to that in cells treated with a control/scrambled siRNA (scr), or in cells treated αvβ3 blocking antibody when compared to cells treated with control isotype antibody (ctrl). (**C**,**D**) cells were then treated without or with PF562271 (PF56) or PF573228 (PF57), two FAK specific inhibitors at the indicated doses. Uptake of glucose analog 2-NBDG was reduced in cells treated with PF56 or PF57. (**E**,**F**) Lactate acid production in the culture medium was decreased in cells treated with FAK inhibitors PF56 or PF57. All experiments were repeated at least three times. Data are represented as mean ± SE, ** *p* < 0.01.

Focal adhesion kinase (FAK) is a signaling molecule activated when integrin receptors bind to extracellular matrix ligands, and FAK plays an essential role in transducing the signaling initiated by integrin engagement with extracellular matrix ligands into cells [27]. We hypothesized that FAK is likely involved in the metabolic shift mediated by αvβ3 and osteopontin engagement; and therefore, FAK inhibition would lead to decreased glucose uptake and glycolysis. To test our hypothesis, we examined whether blockade of FAK activation had an effect on glucose uptake and lactate production in GBM cells plated on osteopontin (Figure 3). FAK activation is decreased in response to siRNA mediated knockdown of αv or β3 when compared to that in cells treated with a control/scrambled siRNA (scr). FAK activation is decreased in cells treated with αvβ3 blocking antibody when compared to cells treated with control isotype antibody (ctrl) (Figure 3A,B and Figure S2). Uptake of 2-NBDG was significantly decreased in LN229 and U251MG cells treated with two FAK specific inhibitors, PF562271 (PF56) or PF573228 (PF57), respectively, in a dose-dependent manner (Figure 3C,D). The lactate levels in the culture medium were also lower in LN229 and U251MG cells treated with FAK inhibitor, PF562271 (PF56) or PF573228 (PF57), in a dose-dependent manner (Figure 3E,F).

**Figure 4:**
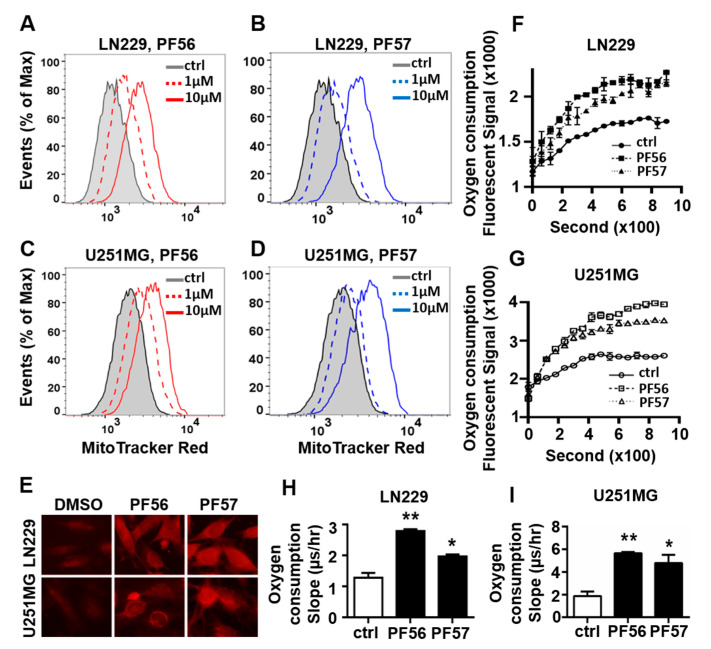
FAK inhibition increases MitoTracker uptake into mitochondria and oxygen consumption in LN229 and U251MG cells. (**A**–**D**) Flow cytometry analysis of MitoTracker stained cells treated with PF573228 (PF57) or PF562271 (PF56) for 16 h at 1 and 10 μM, respectively. (**E**) Representative immunofluorescent images of the MitoTracker labeled LN229 and U251MG in the presence of PF56 or PF57 (400×). (**F**,**G**) Oxygen consumption curve in cells treated with PF56 or PF57 at 10 μM. (**H**,**I**) Cellular oxygen consumption rate (slope) calculated based on the oxygen consumption curve in cells treated with 10 μM FAK inhibitors PF56 or PF57. All experiments were repeated at least three times. Data are represented as mean ± SE, * *p* < 0.05, ** *p* < 0.01.

In addition, FAK inhibition by FAK inhibitor, PF573228 (PF57) or PF562271 (PF56), led to increased fluorescent intensity of MitoTracker labeling in both LN229 and U251MG GBM cells, indicating that FAK inhibition results in an increase in mitochondrial oxidative phosphorylation function (Figure 4A–D). Consistent with the flow cytometry data, microscopic imaging of MitoTracker labeling showed that GBM cells treated with PF573228 or PF562271 appeared stronger mitochondria function (Figure 4E). The cellular oxygen consumption rate, another indicator of mitochondrial oxidative phosphorylation function, was significantly increased in LN229 and U251MG cells treated with FAK inhibitor; PF573228 or PF562271 (Figure 4F–I). Taken together, these results strongly support FAK’s role in the regulation of mitochondrial function in GBM cells, as FAK inhibition decreased glycolysis and increased mitochondrial function likely through disruption of αvβ3 and osteopontin engagement.

### 2.4. Blockade of αvβ3 and Osteopontin Engagement through FAK Inhibition Significantly Decreases Cell Migration and Proliferation in GBM Cells

**Figure 5:**
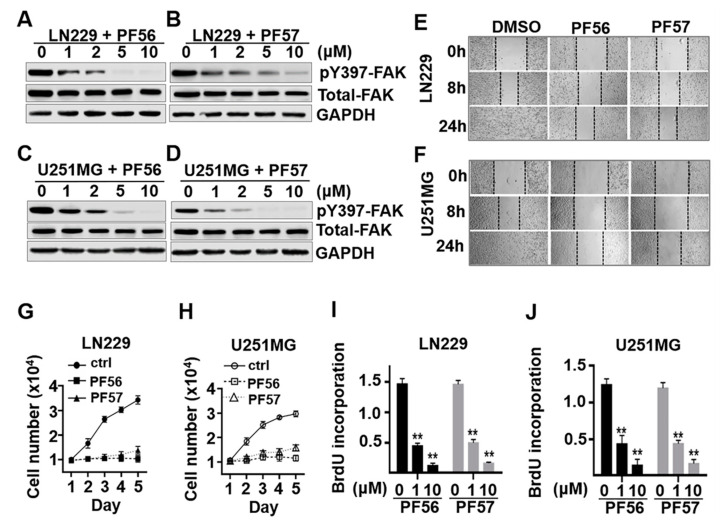
Blockade of FAK activation inhibits migration and proliferation in LN229 and U251MG cells. (**A**–**D**) FAK inhibitor PF562271 (PF56) or PF573228 (PF57), reduced the levels of the active form of FAK (pY397-FAK) in a dose-dependent manner. FAK activation was inhibited over 90% at 10 μM in both cell lines. (**E**,**F**) FAK inhibitors PF56 and PF57 reduced cell migration in a wound scratch heal assays (at 10 μM of PF56 and PF57, 24 h). (**G**,**H**) Cell growth in the presence of 10 μM PF56 or PF57 as measured by direct cell counts. (**I**,**J**) Cell proliferation measured by BrdU proliferation assays in the presence of 10 μM PF56 or PF57. All experiments were repeated at least three times. Data are presented as mean ± SE. ** *p* < 0.01.

FAK activation is essential for integrin αvβ3 and osteopontin engagement in GBM cells, and increased FAK expression and activation have been well documented in GBM [11,28,29,30]. PF562271 (PF56) or PF573228 (PF57) inhibited FAK activation in a dose-dependent manner in LN229 and U251MG cells (Figure 5A–D and Figure S3). Cell migration was determined by wound scratch assays as described previously [31]. Cell migration was significantly inhibited in LN229 and U251MG cells treated with PF562271 or PF573228 when compared to vehicle-treated cells (Figure 5E,F). Furthermore, cell growth was significantly inhibited in cells treated with PF562271 or PF573228 as measured by cell count and BrdU cell proliferation assays (Figure 5G–J). These results suggest that disruption of integrin αvβ3 and osteopontin engagement and FAK inhibition negatively impact glucose metabolism, migration, and growth in GBM cells.

### 2.5. Protein Arginine Methyltransferase -5 (PRMT-5) Regulates Metabolic Shift towards Glycolysis, Migration and Invasion in GBM Cells

PRMT5 belongs to a family of enzymes that transfer the methyl group from S-adenosylmethionine to the arginine side-chains of histones and other proteins [32]. Recent evidence shows that PRMT5 is upregulated in a number of cancers, and PRMT5 is an important enzyme involved in tumorigenesis and stem cell maintenance linked to tumor progression and poor prognosis [33,34,35,36,37]. We have demonstrated that PRMT5 expression is low in normal glial cells and low grade glioma but highly expressed in GBM, and its expression increases with increasing malignant [24]. However, the role of PRMT5 in glucose metabolism in GBM has not been determined.

**Figure 6:**
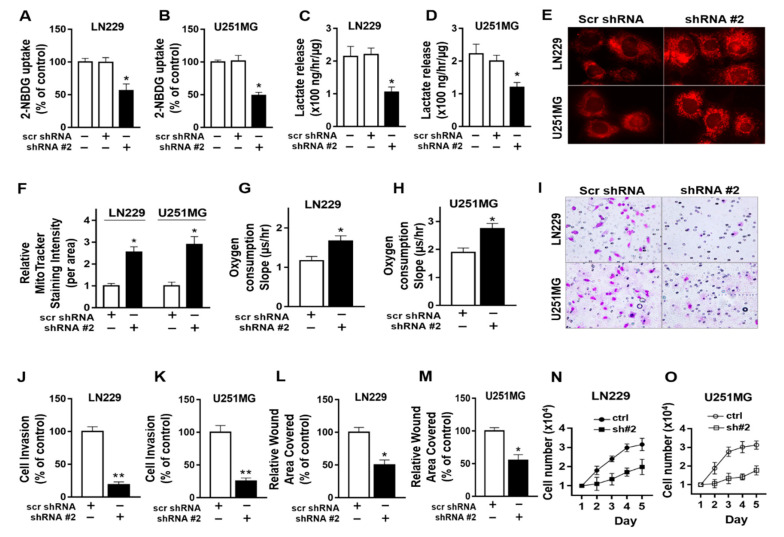
Protein arginine methyltransferase 5 (PRMT5) knockdown inhibits glycolysis, promotes mitochondria function, and decreases invasion and migration in glioblastoma multiforme (GBM) cells. LN229 and U251MG cells were plated on osteopontin (10 μg/mL) coated plates. PRMT5 knockdown by shRNAs was confirmed and published [24]. (**A**,**B**) Uptake of glucose analog 2-NBDG was reduced in cells with PRMT5 knockdown. (**C**,**D**) Lactate levels in culture medium decreased in cells with PRMT5 knockdown. (**E**–**H**) MitoTracker intensity and cellular oxygen consumption rate (slope) were decreased in cells with PRMT5 knockdown, indicating increased mitochondria function. (400× for e) (**I**–**K**) Invasion by Matrigel invasion assays (200× for **I**), (**L**,**M**) cell migration by wound closure assays, and (**N**,**O**) cell growth, were decreased in cells with PRMT5 knockdown. All experiments were repeated at least three times. Data are represented as mean ± SE, * *p* < 0.05, ** *p* < 0.01.

To explore the molecular mechanism associated with the observed metabolic shift upon integrin αvβ3 and osteopontin engagement, the effects of PRMT5 downregulation on glucose metabolism and cell functions were examined in LN229 and U251MG GBM cells plated on osteopontin (Figure 6). PRMT5 downregulation was achieved by PRMT5 specific short hairpin RNA (shRNAs), which we previously described [24]. PRMT5 knockdown by shRNA#2 was confirmed by Western blot analysis (data not shown) and was similar and consistent with our published knockdown data [24]. PRMT5 downregulation by shRNA#2 significantly decreased glucose uptake in LN229 and U251MG cells plated on osteopontin compared to control shRNA treated cells (Figure 6A,B). PRMT5 downregulation significantly decreased the lactate levels in the culture medium in LN229 and U251MG cells compared to control cells (Figure 6C,D). In addition, PRMT5 downregulation significantly increased MitoTracker intensity and cellular oxygen consumption rate in both LN229 and U251GM cells compared to control cells, indicating there was enhanced mitochondria membrane potential and function following PRMT5 downregulation (Figure 6E–G). Functionally, PRMT5 significantly decreased invasion by Matrigel invasion assays (Figure 6H,J), cell migration by wound closure assays (Figure 6K,L), and growth (Figure 6M,N). These results demonstrate that PRMT5 regulates a metabolic shift towards glycolysis and inhibits mitochondria oxidative phosphorylation in GBM cells, and PRMT5 downregulation results in decreased invasion, migration, and growth in GBM cells.

## 3. Discussion

In this study, we showed that the engagement of αvβ3 integrin and osteopontin increases glycolysis and decreases mitochondria oxidative phosphorylation in GBM cells. The metabolic reprogramming was associated with enhanced migration, invasion, and growth of GBM cells mediated by FAK. Blocking αvβ3 function, or knockdown of integrin αv or β3, inhibited the metabolic shift toward glycolysis. Our results support that pharmacological inhibition of FAK, which disrupts the engagement of αvβ3 integrin and osteopontin, reverses the metabolic shift decreases glycolysis, and increases mitochondrial function, and subsequently inhibits migration and growth of GBM cells. We demonstrated PRMT5 also plays an important role in glycolysis and cellular function, evidenced by that PRMT5 downregulation increases mitochondrial function, and decreases glycolysis and inhibits migration, invasion, and growth in GBM cells. Taken together, these results demonstrate a novel role of integrin αvβ3 and osteopontin engagement in metabolic reprogramming in GBM cells. Furthermore, the results indicate that FAK and PRMT5 likely regulate the metabolic reprogramming.

Cancer cells preferentially activate the glycolysis pathway to generate ATP, as well as many metabolic intermediates, needed for biosynthesis of cellular machinery (Warburg effect) [17]. When encountering adverse environments, such as nutrient restriction or growth factor depletion, cancer cells are able to transduce the necessary signals to adjust their metabolic need through the Warburg effect. Reversing the Warburg effect has been investigated as a mechanism for inhibiting or suppressing glioblastoma [38,39,40]. Integrins are important cell surface receptors that interact with their ligands in the tumor microenvironment; at times, they may cooperate with growth factor receptors, such as epidermal growth factor receptor (EGFR) signaling, to accurately transduce environmental cues into the cells [41,42,43]. Because we and others previously demonstrated that osteopontin is important for GBM cell migration and growth [11,14], we examined whether osteopontin and its receptor integrin αvβ3 engagement altered cell metabolism. Our data strongly support that osteopontin and its receptor integrin αvβ3 engagement promotes glycolysis in GBM cells, evidenced by the fact that inhibition of integrin αvβ3 and osteopontin engagement, either by αvβ3 downregulation or using αvβ3 blocking antibody, decreases glucose uptake and increases mitochondrial function, as well as effectively reverses the metabolic shift toward glycolysis.

To understand the downstream signaling of integrin αvβ3 and osteopontin engagement in regulation of the metabolic shift, we examined whether FAK inhibition affects cell metabolism because FAK is a central molecule in integrin receptor mediated signaling, and its role in GBM cell migration, invasion and growth has been well established [23,27,44,45]. FAK signaling has previously been shown to promote glycolysis in other cell types [46,47]. We expect that inhibition of FAK activation blocks the transduction of signaling initiated by integrin αvβ3 and osteopontin engagement. Our experiments using two specific FAK inhibitors provide evidence for the potential downstream mechanism of the metabolic reprogramming mediated by integrin αvβ3 and osteopontin engagement. GBM cells treated with FAK inhibitors showed significantly decreased glucose uptake and increased mitochondrial function in GBM cells plated on osteopontin. The data strongly support that inhibition of FAK blocks the transduction of signaling initiated by integrin αvβ3 and osteopontin engagement, and subsequently reverses the metabolic shift, and inhibits migration, invasion, and growth in GBM cells. We have reported that FAK promotes cell cycle progression through enhancing the cyclins D1 and E in GBM [23]. The current study specifically focuses on the role of integrin αvβ3 and osteopontin engagement on metabolic reprogramming. The exact molecular mechanism by which FAK regulates the metabolic shift toward glycolysis has yet to be determined and is the subject of future investigations.

PRMT5 belongs to a family of enzymes that transfer the methyl group from S-adenosylmethionine to the arginine side-chains of histones and other proteins [32]. Upregulation of PRMT5 is found in a number of cancers, and PRMT5 is considered an important enzyme involved in tumorigenesis and stem cell maintenance and is linked to tumor progression and poor prognosis [33,34,35,36,37]. We have previously reported that PRMT5 expression is up-regulated in GBM and its expression is increased in parallel with malignant progression in GBM [24]. Little is known about the function of PRMT5 in GBM. Here, we explored the potential role of PRMT5 in the metabolic shift of GBM cells upon integrin αvβ3 and osteopontin engagement. PRMT5 downregulation greatly decreases glucose uptake and lactate production, while it increases cellular oxygen consumption rate and mitochondrial function in GBM cells plated on osteopontin. In addition, PRMT5 downregulation results in decreased invasion, migration, and growth in GBM cells. These results demonstrate that PRMT5 promotes metabolic shift toward glycolysis, inhibits mitochondrial oxidative phosphorylation, and promotes migration and invasion in GBM cells. A recent study reports the role of PRMT5 in glycolysis and tumorigenicity in pancreatic cancer [48], and PRMT5 promotes cancer cell migration and invasion [49]. Expression of multiple key and rate limiting enzymes in glycolysis or tricarboxylic acid (TCA) pathway are up- or down-regulated in response to metabolic reprogramming. PRMT5 is recruited to histones through interaction with Schwann Cell Factor 1/positive regulatory domain 4 (SC1/PRDM4) and serves as an epigenetic enzyme essential for maintaining neural stem cells [50].

The study aims to understand the underappreciated role of αvβ3 and osteopontin interaction in glycolysis, and its potential downstream players in GBM. Integrin αvβ3 and osteopontin play critical roles in GBM invasion and progression; importantly, osteopontin is not only expressed in GBM but also in normal brains. Integrin αvβ3 and osteopontin interaction likely plays a more significant role than its interaction with other tumor matrix proteins to facilitates GBM invasion and progression into normal brain areas [11]. The data support that FAK and PRMT5 are a part of downstream signaling events initiated by αvβ3 and osteopontin engagement, and also provides some clues for future studies. The role of FAK in integrin signaling is well supported in literature [27,51]. The link of PRMT5 with αvβ3 or FAK is a new observation but less clear based on limited data in current studies. There are limitations in current studies, but they provide the opportunities for future studies, including how FAK and PRMT5 are linked by αvβ3 and osteopontin engagement, or whether FAK and PRMT5 are independent downstream signaling, or associated, or regulate different components of downstream signaling initiated by αvβ3 and osteopontin engagement. The involvement of PRMT5 is one of new observations which deserves further investigation. We speculate that PRMT5 may be involved in metabolic reprogramming in GBM cells through epigenetic regulation of histone function, for example, through mediating histone arginine methylation and subsequently controlling the expression of key enzymes in the glycolysis or TCA pathway. Future studies will investigate the molecular mechanism by which PRMT5 regulates metabolic shift toward glycolysis in GBM cells. It will be interesting to see if similar signaling can be initiated by other ligands or matrix proteins, or whether other types of tumor cells have similar responses when engaging their particular matrix proteins, or whether non-tumor cells show similar signaling under certain pathological insults. These studies will provide more specific answers regarding to the effect of integrin and matrix engagement on metabolism based on ligand specificity or cell type.

In summary, our studies demonstrate that integrin αvβ3 and osteopontin engagement regulates the metabolic shift toward glycolysis in GBM cells, and downstream FAK activation stimulates glycolysis, while inhibiting mitochondrial oxidative phosphorylation in GBM cells. The metabolic reprogramming co-occurs with increased cell migration, invasion, and growth. It is likely that the reprogramming provides GBM cells with abundant metabolic intermediates in the glycolytic pathway for biosynthesis of cellular building blocks. In addition, reprogramming may generate the needed energy for cell migration, invasion, and growth. Furthermore, PRMT5 regulates the metabolic shift toward glycolysis in GBM cells, which may shed some light on the role of PRMT5 in metabolic reprogramming initiated by integrin and extracellular ligands engagement. These findings provide new insight into how cancer cells regulate their metabolism based on microenvironmental cues transmitted by integrin and extracellular matrix proteins, as well as how the signals eventually translate to metabolic modifications coupled with changes in cell behavior, including migration, invasion, and growth.

## 4. Materials and Methods

### 4.1. Reagent and Antibodies

Mouse anti-αv antibody (Abcam, Cambridge, MA, USA, ab16821), rabbit anti-FAK (Cell Signaling Technology, Danvers, MA, USA, #3285), rabbit anti-phospho FAK Y397 (Cell Signaling Technology #3283), rabbit anti GAPDH (Cell Signaling), Anti-mouse Immunoglobulin G-Horseradish peroxidase (IgG-HRP) and anti-rabbit IgG-HRP (Cell Signaling Technology), FAK inhibitors PF-573228 (Sigma-Aldrich, St. Louis, MO, USA) and PF-562271 (Selleckchem, Houston, TX, USA), Osteopontin (R&D System, Minneapolis, MN, USA, #1433-OP-050), 2-NBDG (Thermo Fisher Scientific, Waltham, MA, USA), Lactate Assay kit (BioVision, Milpitas, CA, USA), Oxygen Consumption Assay Kit (Cayman Chemical, Ann Arbor, MI, USA) and MitoTracker Red CMXRos (Invitrogen, suwanee, FA, USA) were purchased. M-PER (mammalian protein extraction buffer), Halt Protease and Phosphatase Inhibitor Cocktail (100×), bicinchroninic acid (BCA) protein assay kit, and culture medium was purchased from Thermo Scientific. Vectashield HardSet Antifade Mounting Solution was from Vector Laboratories (Burlingame, CA, USA).

### 4.2. Cell Lines and Cell Culture

GBM cell lines LN229 and U251MG were purchased from American Type Culture Collection (ATCC) and Sigma-Aldrich and described previously [23,24]. Cells were maintained on tissue-culture treated plastic dishes at 37 °C, 5% CO_2_, in Dulbecco’s Modified Eagle Medium/Nutrient Mixture F-12 (DMEM/F12) (50/50) media (Corning, Midland, NC, USA) containing 10% fetal bovine serum, supplemented with 2 mM L-glutamine, penicillin and streptomycin. Culture dishes were coated with osteopontin at 10 μg/mL in phosphate-buffered saline (PBS) at 37 °C for 2 h, followed by PBS wash.

### 4.3. Immunoblotting

Immunoblotting was essentially performed as described [52]. Briefly, cultured cells were lysed in M-PER mammalian protein extraction buffer supplemented with 1x Halt proteinase and phosphatase inhibitor cocktail (100 μg/mL aprotinin, 10 μg/mL leupeptin, 2 mg/mL benzolsulfonylfluorid (AEBSF) hydrochloride, 50 μg/mL Bestatin, 200 μg/mL E-64, 100 mg/mL ethylenediamine tetraacetic acid (EDTA), 10 μg/mL Pepstatin A). Protein concentrations were determined by BCA protein assay. Equivalent amounts of 20μg total protein were resolved on a 10% SDS-PAGE gel, transferred to a nitrocellulose membrane, blocked with 5% non-fat milk in tris-buffered saline with tween 20 (TBST), and probed with the primary antibody at 4 °C overnight, followed by a secondary antibody conjugated to horseradish peroxidase. Membrane-bound antibodies were detected by SuperSignal West Dura Chemiluminescent Substrate. Cells were treated with 10 μM PF562271 for 16 h, trypsinized and washed twice in PBS. Cells were gently resuspended in 500 μL PBS and a small portion was collected for BCA protein assay.

### 4.4. BrdU Cell Proliferation Assay and Cell Count

BrdU proliferation assay was performed per manufacturer’s protocol (Millipore, Louis, MO, USA, #2750) and as described [23]. Briefly, LN229 or U251MG cells were seeded at a density of 2 × 10^4^ cells per well in a 96-well plate and treated with anti- αvβ3 antibody (10 μg/mL), FAK inhibitors PF-573228 (10 μM) or PF-562271(10 μM). BrdU was added for the last 16 h of incubation. Cells were then fixed, washed and incubated with anti-BrdU monoclonal antibody and secondary antibody per manufacture’s protocol. After wash, TMB peroxidase substrate was added and incubated for 30 min in the dark, followed by addition of stop solution. Incorporated BrdU was measured by microplate reader at 450 nm (Tecan, Invitrogen). For cell count, LN229 or U251MG cells were seeded at a density of 1 × 10^4^ cells per well in a 96-well plate and treated with FAK inhibitors PF-573228 (10 μM) or PF-562271(10 μM). Cells were trypsinized and viable cells were stained by trypan blue and counted every 24 h after treatment for 5 days.

### 4.5. Wound Closure and Invasion Assays

The migration capabilities of GBM cells were assessed using a scratch wound assay as described [31]. Cells were seeded in 24-well plate and maintained at 37 °C and 5% CO_2_ for overnight to form a confluent monolayer. Then, a linear wound was generated in the monolayer with a sterile plastic pipette tip. Any cellular debris was removed by washing with PBS. Wound closure was monitored by collecting digitized images at 0, 8, and 24 h post scratch, respectively. In vitro invasion assay was performed with the kit with Matrigel-coated inserts according to the manufacturer’s instructions (BD Biosciences, San Jose, CA, USA) as described [53]. Then, 1 × 10^5^ cells/well was added to the upper compartments of the invasion chamber. The values obtained were calculated using the number of invaded cells from three filters after 24 h. Final results were pooled from two to three individual experiments.

### 4.6. Glucose Uptake Assay

Cellular glucose uptake was measured by determining the amount of 2-NBDG uptake by the cells normalized against cellular protein, according to previous report [54]. Briefly, cells were seeded in a 96-well plate at 1.5 × 10^4^ cells/well in 100 µL culture medium. After treatment, the growth medium was then replaced by 150 μL of starvation medium (glucose and fetal bovine serum (FBS) free supplemented with 1% bovine serum albumin (BSA)). After 1-h starvation, the medium was replaced with fresh starvation medium supplemented with 50 μM 2-NBDG and drug treatment accordingly, and incubated at 37 °C for 1 h. Medium was carefully aspirated, and cells were washed twice with PBS; then, 100 µl of PBS was added to each well, and the plate was read immediately (excitation/emission = 465/540 nm) in a microplate reader (Tecan, Invitrogen). Subsequently, cellular protein from each well was determined by BCA assay. The amount of 2-NBDG taken up by cells were normalized against total cellular protein from each well.

### 4.7. Mitochondrial Activity Assay

MitoTracker Red CMXRos is a lipophilic cationic fluorescent dye which accumulates in active mitochondria in a manner dependent on the mitochondrial membrane potential as described [25,26]. Mitochondrial activity was evaluated by measuring fluorescence levels upon staining with MitoTracker Red CMXRos at 100 nM for 20 min at 37 °C. For fluorescence imaging, glioblastoma cells with different treatments were incubated with 100 nM MitoTracker Red CMXRos for 20 min at 37 °C. Cells were then washed twice with ice-cold PBS, and fixed in 3.7% paraformaldehyde for 15 min on ice. After two washes with PBS, cells were mounted with Vectashield HardSet Antifade Mounting Solution and imaged by an Olympus microscope (Center Valley, PA, USA) equipped with a digital camera. For flow cytometry analyses, treated cells were incubated with 100 nM MitoTracker Red CMXRos for 20 min at 37 °C. After incubation, cells were trypsinized, washed twice with PBS, and fixed in 3.7% paraformaldehyde in for 15 min. Cells were then immediately analyzed by LSRII analyzer (BD BioSciences, San Jose, CA, USA).

### 4.8. Measurement of Extracellular Oxygen Consumption Rate

Mitochondria oxygen consumption was measured according to manufacturer’s manual. Briefly, cells were seeded in a black, clear bottom 96-well plate in 200 μL of culture medium. After indicated treatments, the number of cells plated in each treatment conditions were adjusted such that the total number of cells were 4 × 10^4^ cells/well at the time of measurement. After a 16 h incubation, culture medium was replaced with 150 μL fresh medium with the according drugs. MitoXpress Xtra Solution (10 μL) was added to each well, and then 100 μL warm mineral oil was gently overlayed on top to seal off oxygen supply. Fluorescence was immediately measured at excitation/emission = 380/650 with a delay time of 30 μs and integration time of 100 μs using a pre-warmed temperature controlled (37 °C) plate reader (Tecan Infinite 200, Invitrogen). Oxygen consumption was indicated by increases in fluorescence signals. Oxygen consumption rates were assessed by calculating the slope of the linear regions of the curves.

### 4.9. Lactate Assay

Lactate was measured in 96-well plate according to manufacturer’s manual (BioVision). Culture medium was collected from cells after different treatments, and deproteinated by incubation with 250 μM metaphosphoric acid (MPA) (Cayman #700518) for 5 min on ice, followed by neutralization with potassium carbonate. Standard curves were constructed using solutions containing known concentrations of lactate. Lactate was determined by measuring fluorescence at Ex/Em = 535/587 nm using a microplate reader and normalized against total cellular protein (Tecan, Invitrogen). Total cell mass from each well was determined by BCA assay, and the amount of lactate released into medium was evaluated by normalizing lactate against total cell mass from each well.

### 4.10. Statistical Analysis

Data analysis and graph preparations were made by Prism GraphPad 7. Two-tailed Student’s *t* tests and ANOVA were used to determine the statistical significance between two or more groups (GraphPad, San Diego, CA, USA) and were expressed as mean + standard error (SE). All experiments were repeated at least three times. A *p* value < 0.05 was considered to be statistically significant.

## 5. Conclusions

Integrin αvβ3 and osteopontin engagement plays an important role in promoting the metabolic shift toward glycolysis and inhibiting mitochondrial oxidative phosphorylation in glioblastoma cells. The metabolic shift in cell energy metabolism is coupled with increased migration, invasion, and growth. In addition, FAK and PRMT5 regulates the metabolic shift in glioblastoma cells plated on osteopontin. These findings provide new insights into how cancer cells regulate their metabolism based on microenvironmental cues transmitted by integrin and extracellular matrix ligands.

## Data Availability

Data presented in this study are available in the article and in supplementary materials.

## Supplementary Materials

The following are available online at https://www.mdpi.com/2072-6694/13/5/1111/s1, Figure S1: Representative Western blot and quantification of siRNA mediated knockdown efficiency in LN229 and U251MG cells. Figure S2: Representative Western blot and quantification of the effects of downregulation of αv and β3 on FAK activation in LN229 and U251MG cells; and effects of blocking αvβ3 on FAK activation in LN229 and U251MG cells. Figure S3: Representative Western blot and quantification of the effect of PF56 and PF57 on inhibition of FAK activation in a dose dependent manner in LN229 and U251MG cells.
