# Supplementary figures and images for: Integrin αvβ3 Engagement Regulates Glucose Metabolism and Migration through Focal Adhesion Kinase (FAK) and Protein Arginine Methyltransferase 5 (PRMT5) in Glioblastoma Cells
Source: Cancers (Basel). 2021 Mar 5;13(5):1111. doi: 10.3390/cancers13051111 (PMC7961489; doi:10.3390/cancers13051111)

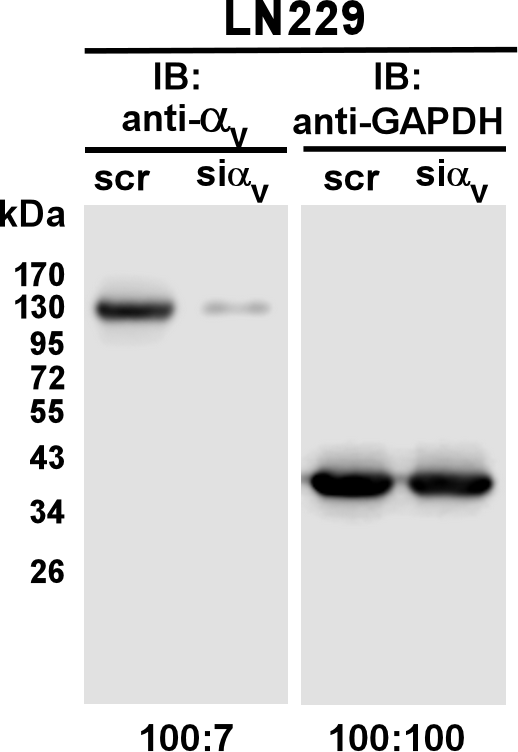

Supplement: Supplementary file 1 [file cancers-13-01111-s001.zip › WB figures/Fig1-LN229-aV.tif]

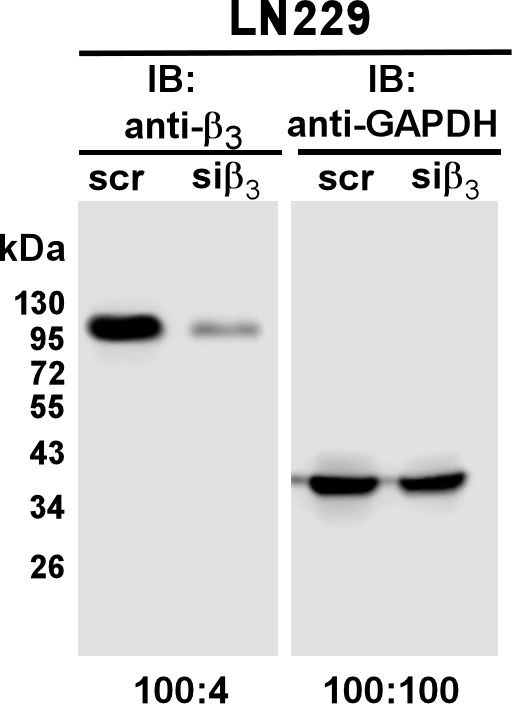

Supplement: Supplementary file 1 [file cancers-13-01111-s001.zip › WB figures/Fig1-LN229-b3.tif]

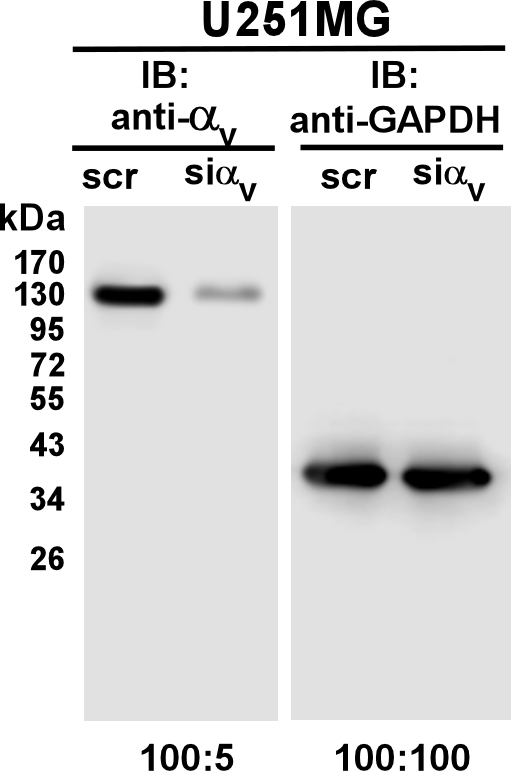

Supplement: Supplementary file 1 [file cancers-13-01111-s001.zip › WB figures/Fig1-U251MG-aV.tif]

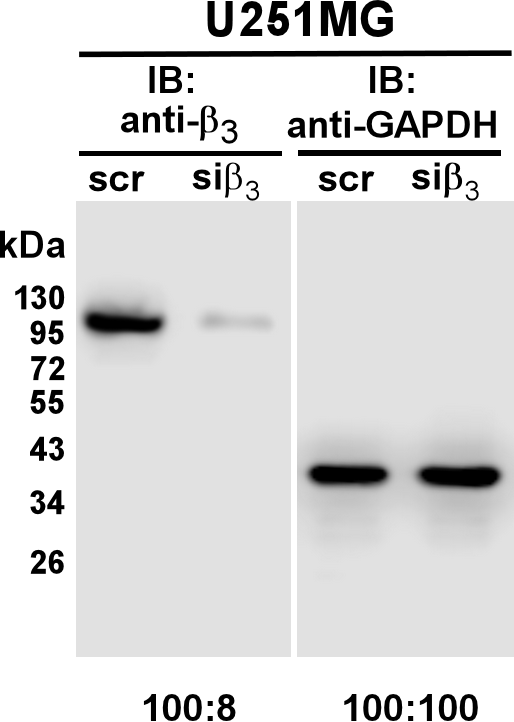

Supplement: Supplementary file 1 [file cancers-13-01111-s001.zip › WB figures/Fig1-U251MG-b3.tif]

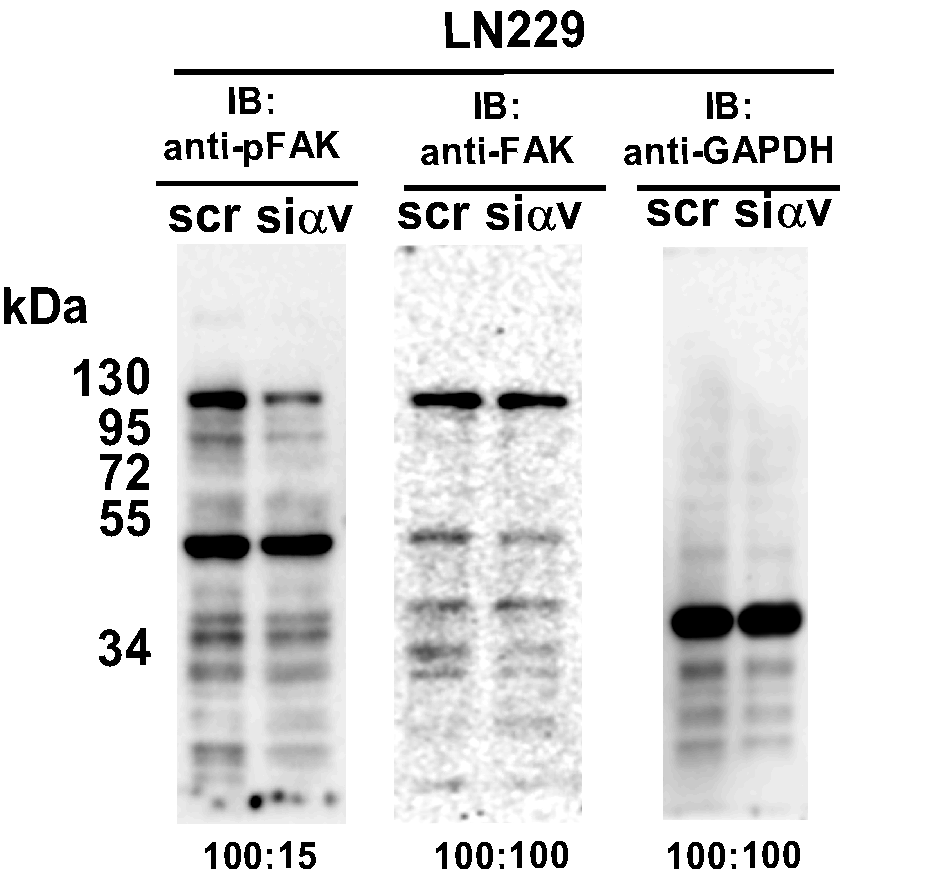

Supplement: Supplementary file 1 [file cancers-13-01111-s001.zip › WB figures/Fig3A-LN229--aV.tif]

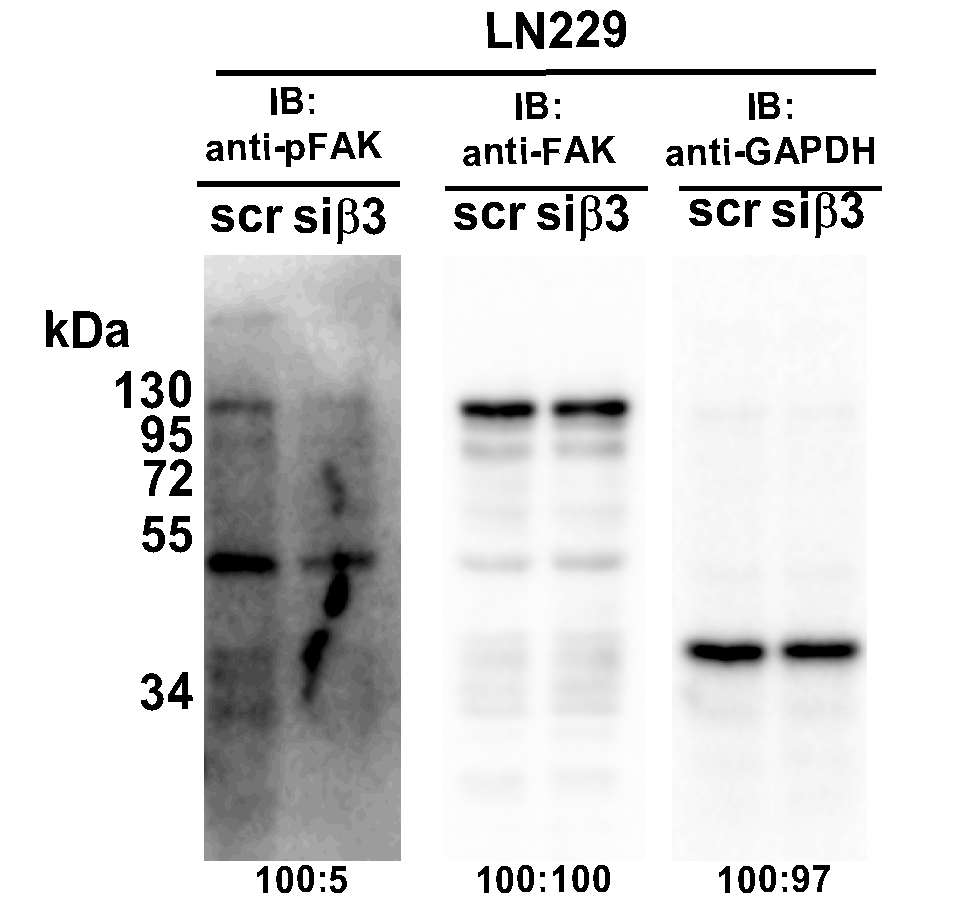

Supplement: Supplementary file 1 [file cancers-13-01111-s001.zip › WB figures/Fig3A-LN229-b3.tif]

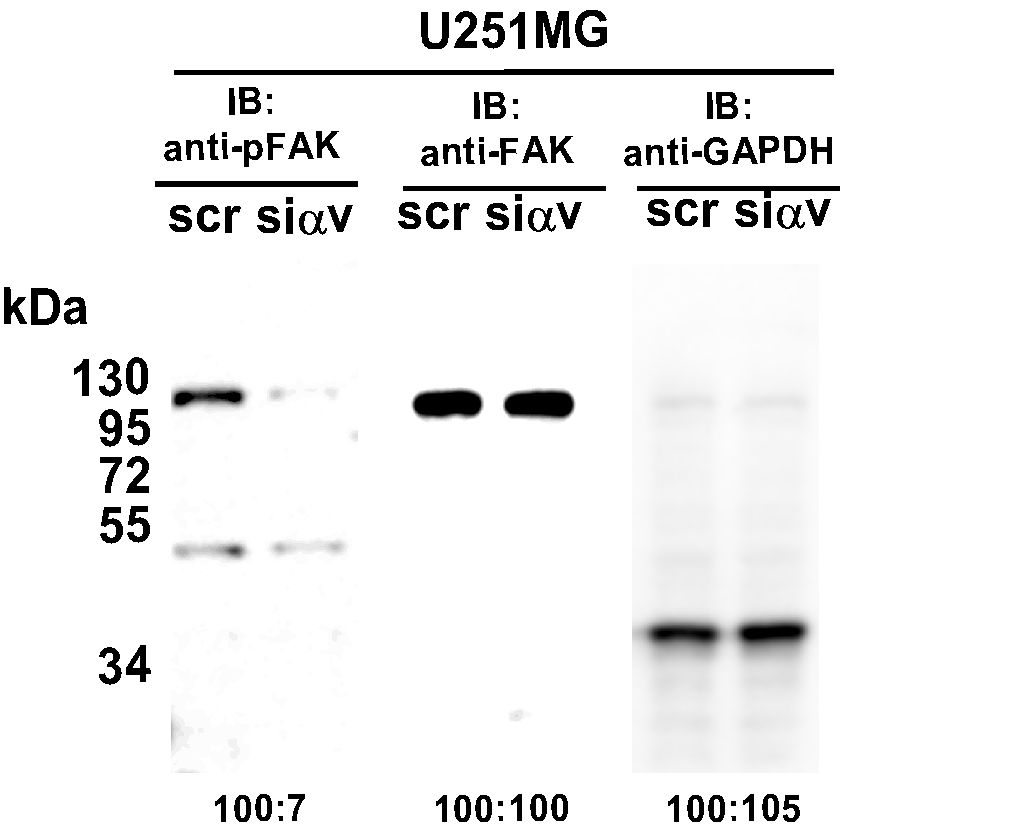

Supplement: Supplementary file 1 [file cancers-13-01111-s001.zip › WB figures/Fig3A-U251MG-aV.tif]

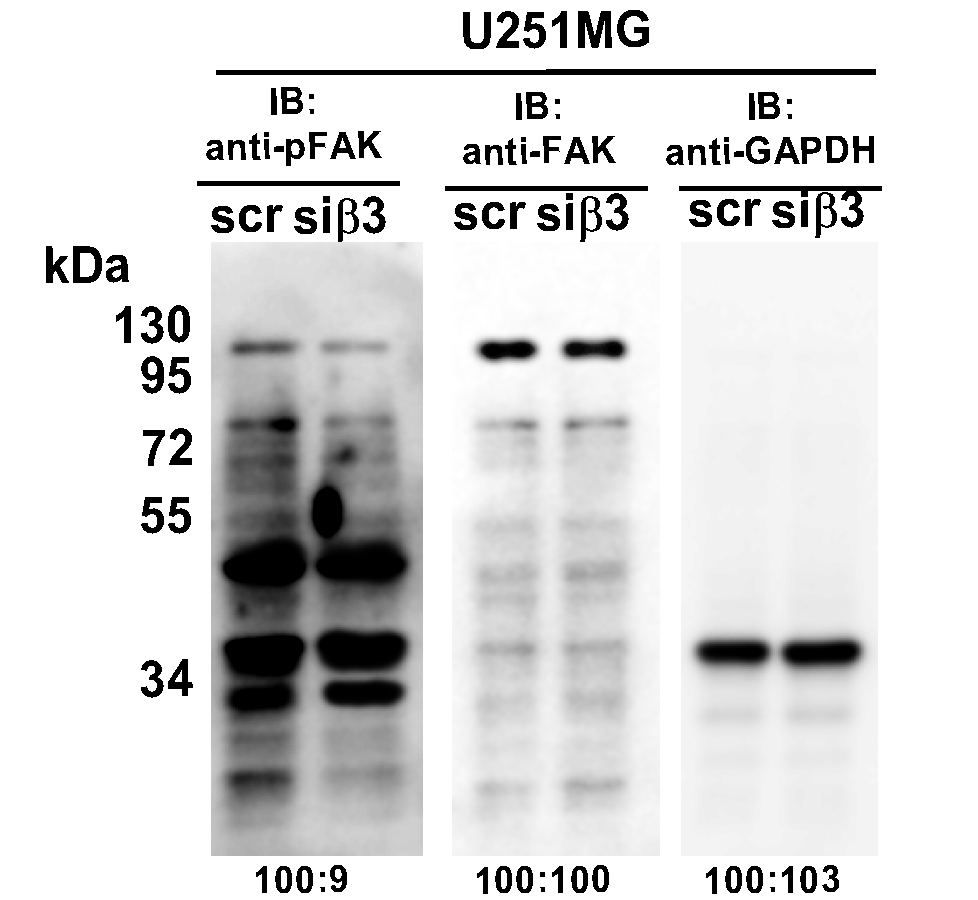

Supplement: Supplementary file 1 [file cancers-13-01111-s001.zip › WB figures/Fig3A-U251MG-b3.tif]

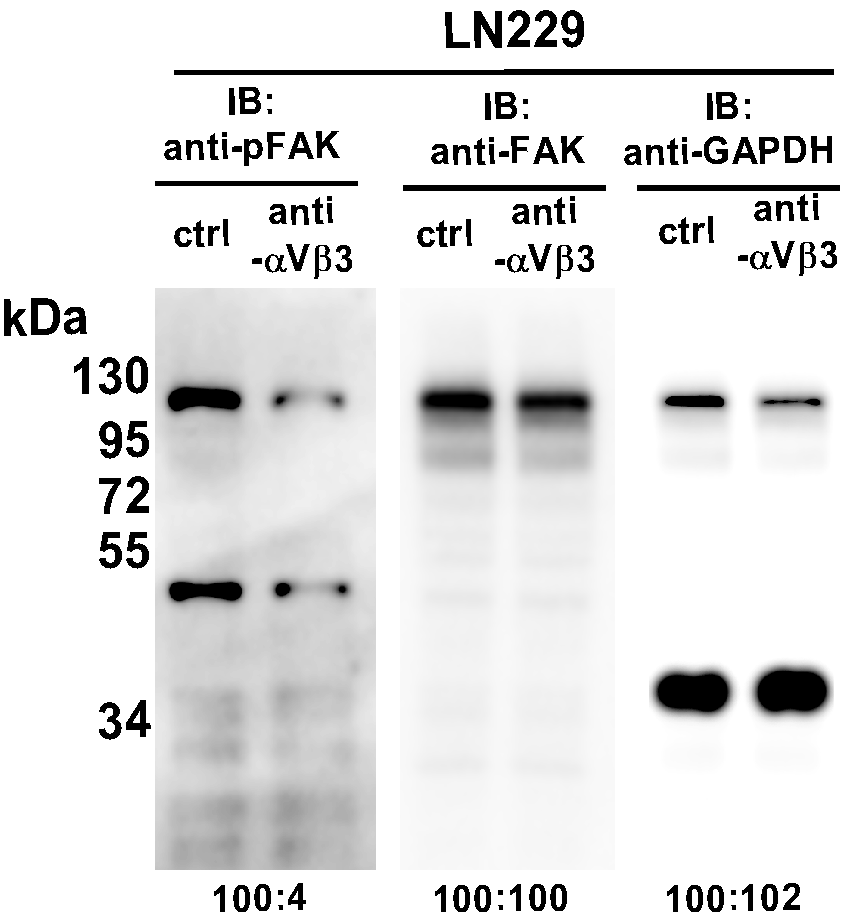

Supplement: Supplementary file 1 [file cancers-13-01111-s001.zip › WB figures/Fig3B-LN229-aVb3.tif]

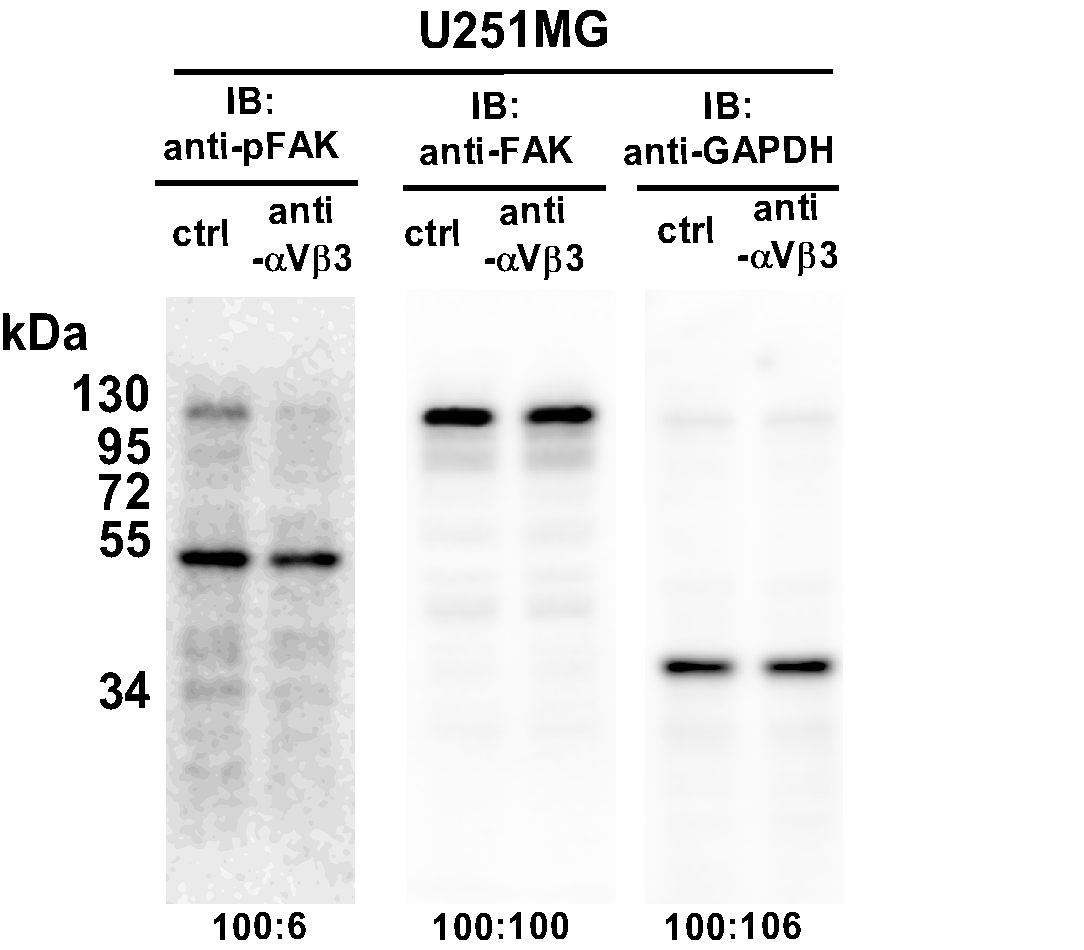

Supplement: Supplementary file 1 [file cancers-13-01111-s001.zip › WB figures/Fig3B-U251MG-aVb3.tif]

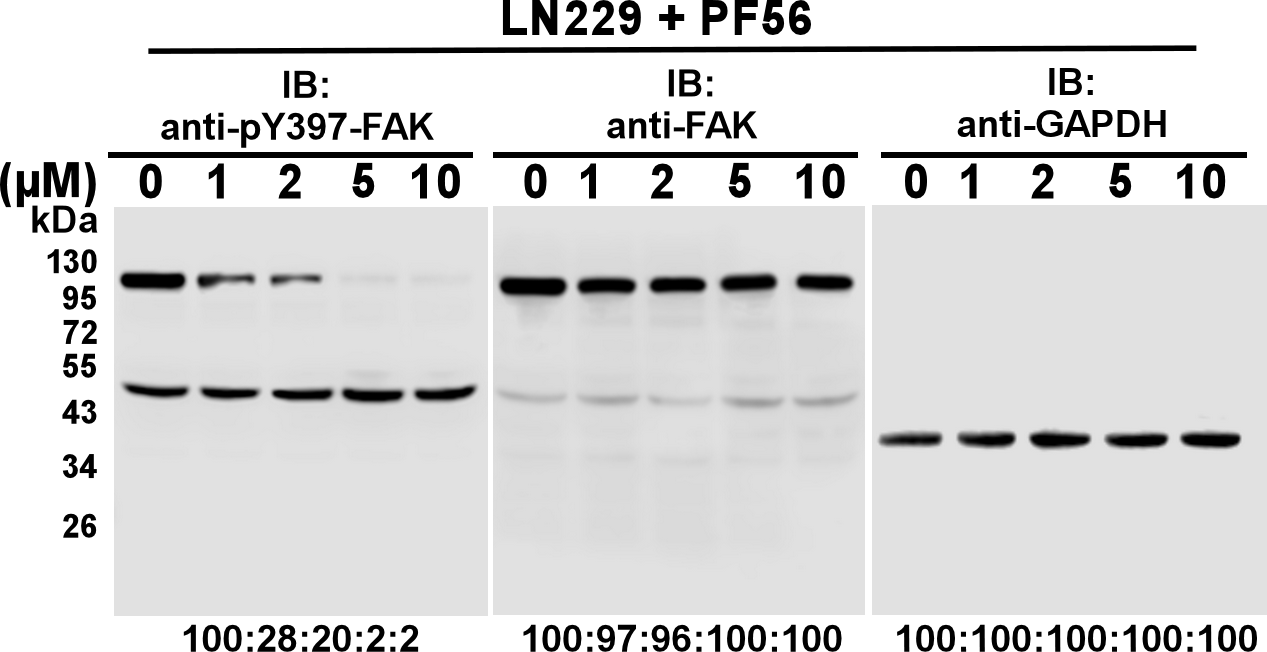

Supplement: Supplementary file 1 [file cancers-13-01111-s001.zip › WB figures/Fig5A.tif]

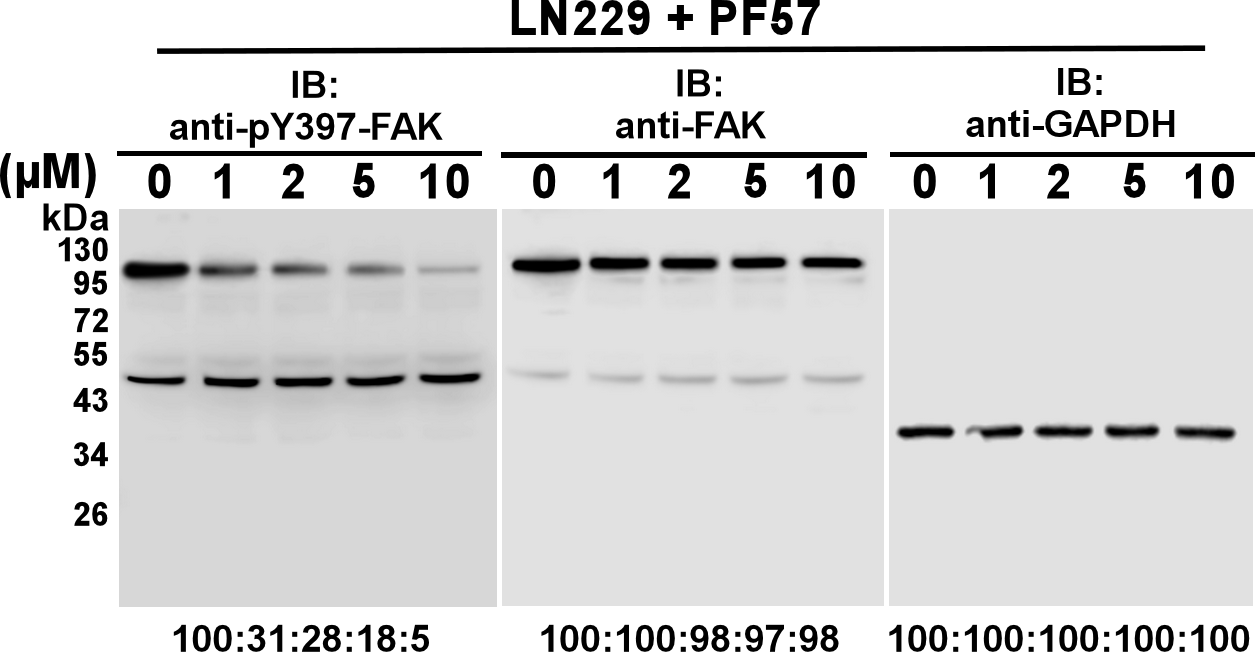

Supplement: Supplementary file 1 [file cancers-13-01111-s001.zip › WB figures/Fig5B.tif]

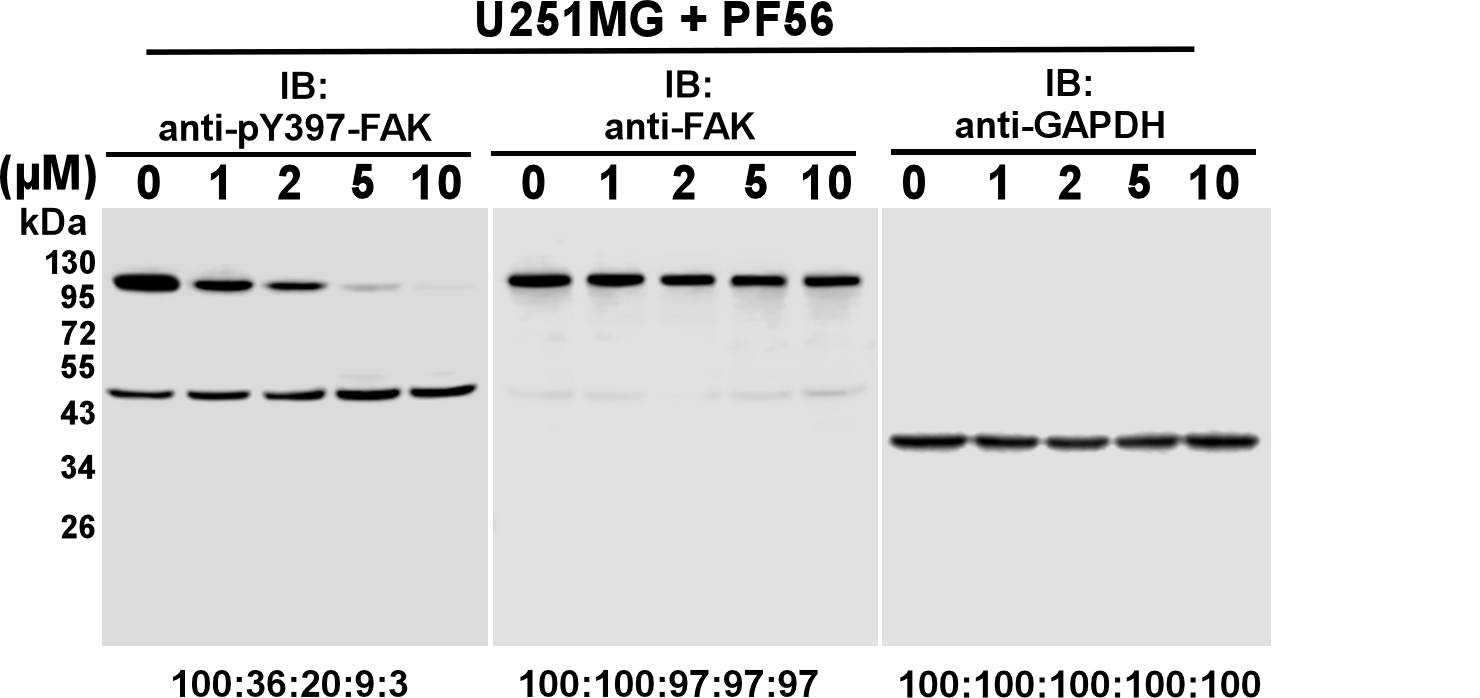

Supplement: Supplementary file 1 [file cancers-13-01111-s001.zip › WB figures/Fig5C.tif]

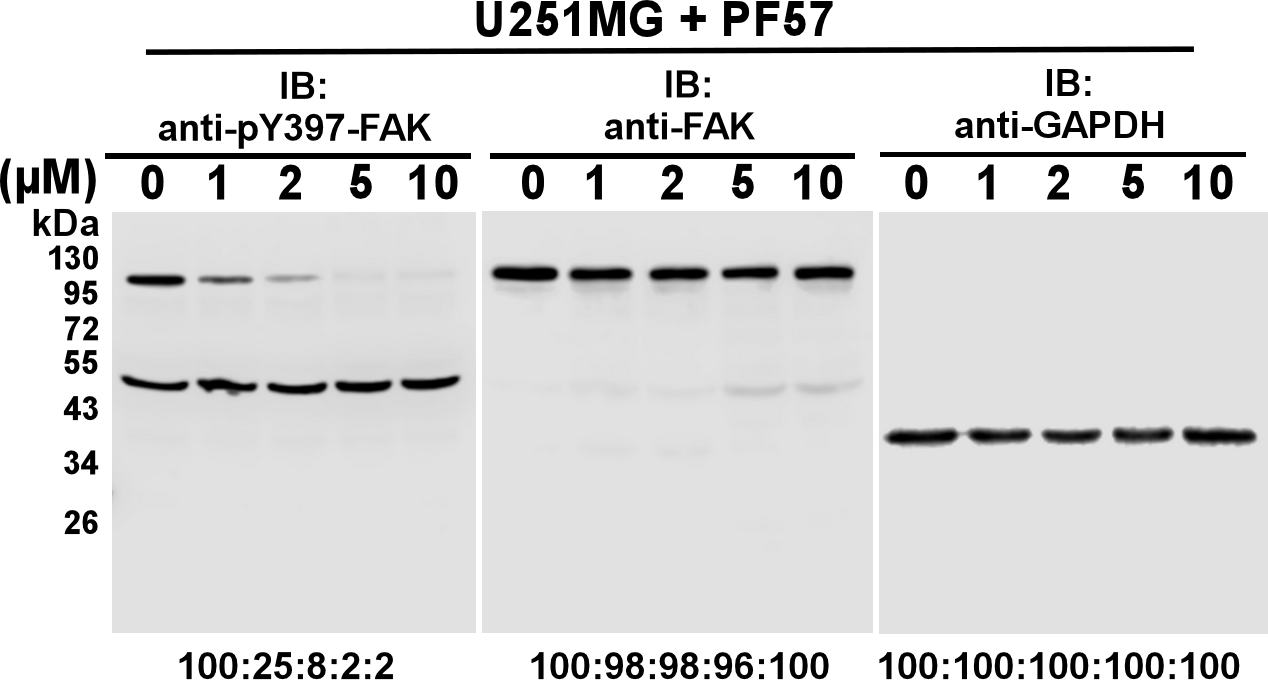

Supplement: Supplementary file 1 [file cancers-13-01111-s001.zip › WB figures/Fig5D.tif]
